# Extraction of High-Precision Urban Impervious Surfaces from Sentinel-2 Multispectral Imagery via Modified Linear Spectral Mixture Analysis

**DOI:** 10.3390/s18092873

**Published:** 2018-08-31

**Authors:** Rudong Xu, Jin Liu, Jianhui Xu

**Affiliations:** 1 State Key Laboratory of Information Engineering in Surveying, Mapping, and Remote Sensing, Wuhan University, Wuhan 430079, China; xurudong@139.com; 2 Guangzhou Institute of Geography, Guangzhou 510070, China; xujianhui306@163.com; 3 Key Laboratory of Guangdong for Utilization of Remote Sensing and Geographical Information System, Guangzhou 510070, China; 4 Guangdong Open Laboratory of Geospatial Information Technology and Application, Guangzhou 510070, China

**Keywords:** Sentinel-2, modified linear spectral mixture analysis, normalized difference built-up index, normalized difference vegetation index, urban impervious surface

## Abstract

This study explores the performance of Sentinel-2A Multispectral Instrument (MSI) imagery for extracting urban impervious surface using a modified linear spectral mixture analysis (MLSMA) method. Sentinel-2A MSI provided 10 m red, green, blue, and near-infrared spectral bands, and 20 m shortwave infrared spectral bands, which were used to extract impervious surfaces. We aimed to extract urban impervious surfaces at a spatial resolution of 10 m in the main urban area of Guangzhou, China. In MLSMA, a built-up image was first extracted from the normalized difference built-up index (NDBI) using the Otsu’s method; the high-albedo, low-albedo, vegetation, and soil fractions were then estimated using conventional linear spectral mixture analysis (LSMA). The LSMA results were post-processed to extract high-precision impervious surface, vegetation, and soil fractions by integrating the built-up image and the normalized difference vegetation index (NDVI). The performance of MLSMA was evaluated using Landsat 8 Operational Land Imager (OLI) imagery. Experimental results revealed that MLSMA can extract the high-precision impervious surface fraction at 10 m with Sentinel-2A imagery. The 10 m impervious surface map of Sentinel-2A is capable of recovering more detail than the 30 m map of Landsat 8. In the Sentinel-2A impervious surface map, continuous roads and the boundaries of buildings in urban environments were clearly identified.

## 1. Introduction

With rapid urbanization, urban impervious surfaces have been greatly expanded, which has led to a decrease in the area of pervious surfaces, including forests, green spaces, bare soils, and wetlands [1]. Urban impervious surfaces mainly include roads, streets, highways, rooftops, and sidewalks, which restrict precipitation from directly infiltrating into the ground. This may result in a high risk of urban rainstorm waterlogging from uncontained surface runoff, as well as increase the urban heat island effect [1,2,3]. Therefore, urban impervious surfaces have become an important indicator of urban rainstorm waterlogging, solar energy balance, and non-point-source water pollution in both environmental and socioeconomic studies, focused on measuring, urban growth, and estimating population distributions [4,5,6,7].

In recent years, images at many different spatial resolutions from satellite-based sensors have been evaluated and applied to estimate impervious surfaces, including Landsat Thematic Mapper/Enhanced Thematic Mapper Plus (TM/ETM+) [8,9,10,11], Landsat 8 Operational Land Imager (OLI) [1,12,13], Moderate Resolution Imaging Spectroradiometer (MODIS) [14], IKONOS [15,16], QuickBird [17], Advanced Spaceborne Thermal Emission and Reflection Radiometer (ASTER) [18,19], GaoFen-1 (GF-1) [20], Synthetic Aperture Radar (SAR) data [21,22,23], and nighttime light data [24]. Furthermore, numerous studies have also been conducted with applications fusing multi-source remote sensing imageries to estimate impervious surfaces, containing the fusion of SAR/InSAR and optical data, Defense Meteorological Satellite Program’s Operational Linescan System (DMSP-OLS) data and MODIS/Landsat data, LiDAR, and high-resolution digital aerial orthoimages [25,26,27,28,29,30].

Depending upon the research objectives, many methods have been proposed to extract impervious surfaces using different remote sensing images [31,32,33,34]. These methods are mainly divided into pixel- and subpixel-scale methods [26,35,36,37,38]. Due to its rich spatial detail for impervious surface mapping, pixel-scale methods have often been applied to high spatial resolution images to map urban impervious surfaces [16,17,39]. However, mapping impervious surfaces is influenced by shadows from high spatial resolution urban images [38], such that it is difficult to clearly distinguish impervious surfaces from vegetation. Additionally, many mixed pixels, containing both impervious surface and vegetation information, may still be found in the high spatial resolution images because tree canopies, especially along roads, can be completely occlusive [17]. Therefore, pixel-scale methods may underestimate impervious surface areas in urban areas [40].

In comparison with traditional pixel-scale methods, some subpixel scale methods, commonly linear spectral mixture analysis (LSMA), have been shown to be superior at mapping impervious surfaces. Linear spectral mixture analysis (LSMA) [18,36,41,42] has been widely applied in low to medium resolution and hyperspectral remote sensing images to quantitatively extract the impervious surface fractions in mixed pixels at a subpixel scale. However, the LSMA may still have difficulty in extracting high-precision impervious surfaces, because of the similar spectral reflectance among different land cover types. Thus, modified LSMA (MLSMA) methods have been proposed to improve the accuracy of impervious surface extractions [43,44,45,46]. Xu et al. [1] proposed a MLSMA method by integrating a normalized difference built-up index (NDBI) and normalized difference vegetation index (NDVI). The MLSMA has been used to extract 30 m urban impervious surface fractions in the city of Guangzhou, China, with the Landsat 8 OLI images from 18 October 2015. The experimental results showed that the MLSMA could extract high-precision impervious surface fraction. However, the modified LSMA has not been applied to higher spatial resolution remote sensing images for extracting impervious surfaces in urban areas at a finer scale. For example, the newly launched Sentinel-2 satellites can provide 10 m and 20 m remote sensing images, which have received little attention in investigations of urban impervious surfaces.

Sentinel-2 is an Earth observation mission developed by the European Space Agency (ESA) for fine spatial resolution global monitoring, including forest monitoring, detecting changes in land cover, and natural disaster management [47,48,49,50]. Sentinel-2 consists of two identical satellites, namely Sentinel-2A and Sentinel-2B, which were launched in June of 2015 and 7 March 2017, respectively. The Sentinel-2 Multispectral Imager (MSI) provides 13 spectral bands in the visible, near infrared and shortwave infrared parts of the spectrum [47] (Table 1). Sentinel-2A and -2B can together revisit the same region every 5 days. The Sentinel-2 data have been successfully applied to vegetation monitoring, the mapping of water bodies and cropland, and the monitoring of urban areas [51,52,53,54]. However, the Sentinel-2 data have not been used to extract urban impervious surface fraction. Great effort should be made in extracting urban impervious surface fractions at higher spatial resolutions (e.g., at the subpixel scale) to improve our understanding of the effects of impervious materials on urban environments. In this study, MLSMA was applied to a high-resolution Sentinel-2A image to extract the impervious surface, vegetation, and soil fractions in the main region of Guangzhou. We aimed to improve the extraction accuracy of urban impervious surface fractions at a finer scale. The study area and Sentinel-2A image data are introduced in Section 2. Section 3 gives a detailed description of the automatic extraction of built-up and MLSMA methods. Section 4 presents the experimental results and an accuracy assessment. A discussion and concluding remarks are presented in Section 5 and Section 6.

## 2. Study Area and Data

### 2.1. Study Area

**Figure 1:**
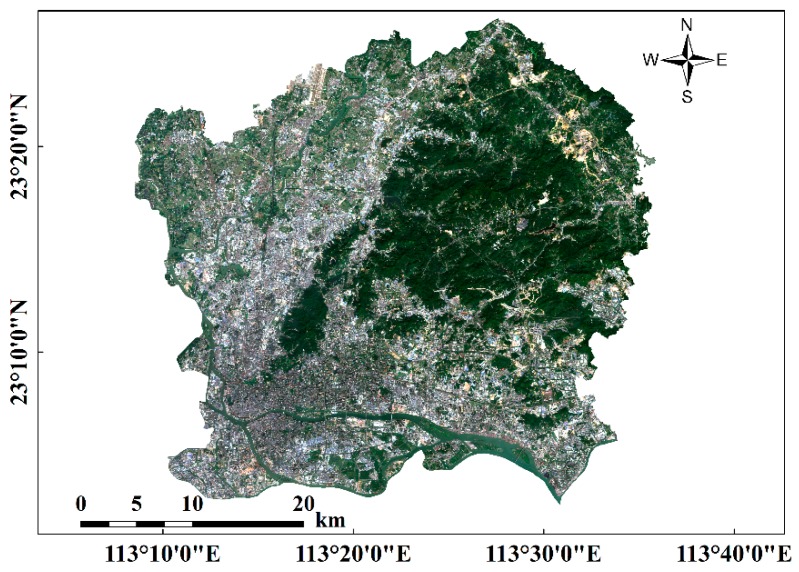
Sentinel-2A RGB image (R: band 4, G: band 3, B: band 2) of the study area.

The city of Guangzhou (22°26′ N–23°56′ N, 112°57′ E–114°30′ E), which is located in south-central Guangdong in southern China, is the political, economic, and cultural center of the province of Guangdong. Guangzhou contains nine administrative districts and two county-level cities. The central area of Guangzhou was selected as the study area, and includes the districts of Liwan, Yuexiu, Haizhu, Tianhe, and Huangpu (Figure 1). The study area has a subtropical monsoon climate, with a mean temperature of 20–22 °C and abundant rainfall. The annual rainfall reaches 1720 mm, most of which occurs from April to June due to the East Asian Monsoon [1]. Due to the high impervious areas, the study area may be affected by rainstorm waterlogging.

### 2.2. Sentinel-2A Image

The Sentinel-2A Level-1C (L1C) product was used in this study, which was produced by radiometric and geometric corrections. The L1C product provides the top of atmosphere (TOA) reflectance. One scene of the Sentinel-2A L1C image with cloud cover of 0% acquired on 1 November 2017 was downloaded from Sentinels Scientific Data Hub (https://scihub.copernicus.eu/). The Level-2A (L2A) surface reflectance was derived through the Sen2Cor Atmospheric Correction Processor, which was integrated into the Sentinel Application Platform (SNAP) version 5.0. The L2A reflectance products were geometrically rectified to the Universal Transverse Mercator (UTM) projection system (zone 49 N). The L2A images of bands 11 and 12 with a resolution of 20 m were resampled to 10 m using the nearest neighbor interpolation. Six bands of Sentinel-2A L2A reflectance products (Table 1, Bands 2, 3, 4, 8b, 11 and 12) were used for extracting impervious surface fractions.

## 3. Methods

**Figure 2:**
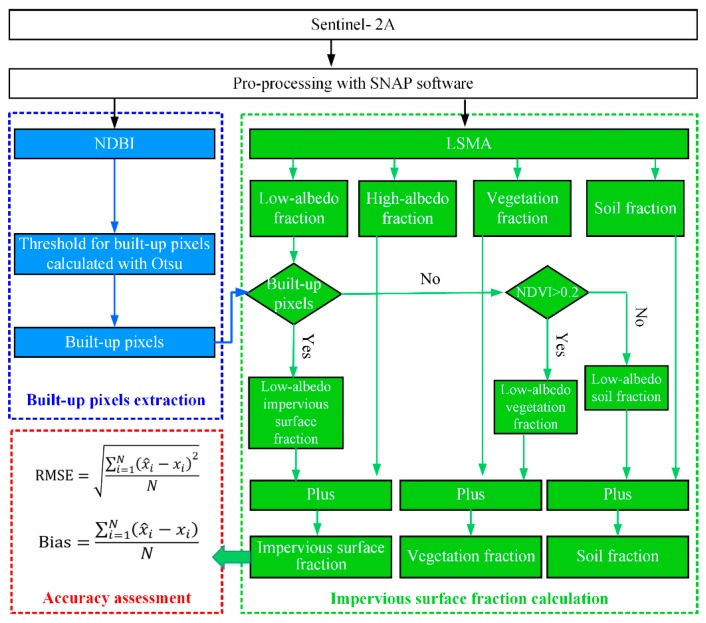
Flow chart for the extraction of impervious surface fraction in this study. LSMA: linear spectral mixture analysis; NDVI: normalized difference vegetation index; NDBI: normalized difference built-up index.

The extraction process for impervious surface fraction based on the Sentinel-2A L2A reflectance product contained three major steps: (1) extracting the built-up pixels automatically via Otsu’s method; (2) extracting fraction maps of high-albedo, low-albedo, vegetation, and soil using LSMA; and (3) modifying the fractions of impervious surface, vegetation, and soil by combining the built-up image and NDVI. The detailed process of impervious surface extraction is shown in Figure 2.

### 3.1. Automatic Extraction of Built-Up

The NDBI, which takes advantage of the unique spectral characteristic of built-up areas and other land cover types, has been widely used to extract built-up areas from remote sensing imagery. The NDBI was used to automate the process of mapping built-up areas by integrating Otsu’s method. The NDBI was calculated using the near-infrared band (NIR) and shortwave infrared band 1 (SWIR1) as follows [55]:

(1) $$NDBI=\frac{\rho_{SWIR1,}-\rho_{NIR}}{\rho_{SWIR1}+\rho_{NIR}}$$

where $\rho_{SWIR1}$ and $\rho_{NIR}$ are the reflectances of band 11 and band 8 of Sentinel-2A imagery, respectively.

In this study, based on the NDBI, the built-up pixels were identified using Otsu’s method. In general, the built-up pixels were extracted by setting an NDBI value greater than 0. However, previous studies have demonstrated that most pixels with an NDBI value less than 0 may belong to built-up pixels [6,56]. Xu, Zhao, Zhong, Zhang, Liu, and Sun [6] have also shown that artificially determined NDBI threshold values may lead to large errors in extracting built-up areas. Otsu’s method [57] uses the maximum interclass variance criterion to find an optimal threshold of an image, and has been successfully utilized for extracting water and land pixels [58,59,60]. To improve the accuracy of the built-up area extraction, Otsu’s method was used to automatically extract built-up areas. The mathematical formula for Otsu’s method is as follows [57]:

(2) $$\{\begin{array}{c} \sigma^{2}=P_{nu,}\cdot {\left(M_{nu}-M\right)}^{2}+P_{u}\cdot {\left(M_{u}-M\right)}^{2}\\ M=P_{nu}\cdot M_{nu}+P_{u}\cdot M_{u}\\ \begin{array}{c} P_{nu}+P_{u}=1\\ t=arg\;\underset{a\leq t\leq b}{\max}\left[P_{u}\cdot P_{nu}\cdot {\left(M_{u}-M_{nu}\right)}^{2}\right] \end{array} \end{array}$$

where σ is the interclass variance, *M* is the mean value of the NDBI image, $P_{nu}$ and $P_{u}$ are the percentages of non-built-up and built-up pixels, respectively, $M_{nu}$ and $M_{u}$ are the mean values of non-built-up and built-up pixels of the NDBI image, respectively, and *t* is the optimal threshold. Otsu’s method was applied to the NDBI image to automatically obtain the built-up pixels. In this study, Otsu’s method was implemented using the binary thresholding function in the ArcGIS software package, version 10.5. In the built-up image, pixels with a value of 1 were expressed as built-up pixels, and other pixels with a value of 0 were expressed as non-built-up pixels. 

### 3.2. Modified Linear Spectral Mixture Analysis

In this study, a MLSMA method proposed by Xu et al. [1] was applied to the Sentinel-2A image to extract the fractions of impervious surface, vegetation, and soil. Firstly, the water body of the Sentinel-2A image was masked by the modified normalized difference water index (MNDWI) [61].

(3) $$MNDWI=\frac{\rho_{Green,}-\rho_{SWIR1}}{\rho_{Green}+\rho_{SWIR1}}$$

where $\rho_{Green}$ and $\rho_{SWIR1}$ are the reflectances of bands 3 and 11 of Sentinel-2A imagery, respectively. The fractions of high-albedo, low-albedo, vegetation, and soil endmembers were then calculated using conventional linear spectral mixture analysis (LSMA) as follows [62]:

(4) $$R_{i}=\sum_{k=1,}^{n}f_{k}R_{ik}+\varepsilon_{i}$$

where *k =* 1, 2, …, *n*, *n* is the number of endmembers, *i =* 1, 2, …, *m*, *m* is the number of spectral bands, *R_i_* is the spectral reflectance of band *i* of a mixed pixel, *f_k_* is a fraction of endmember *k* within the mixed pixel, *R_ik_* is the known spectral reflectance of endmember *k* within a mixed pixel of band *i* and $\varepsilon_{i}$ is the error of band *i*. Four endmembers, such as high-albedo, low-albedo, vegetation, and soil endmembers, were determined in this study. The spectral reflectance of six bands from the Sentinel-2A image (Table 1) were used as inputs of LSMA to estimate the fraction of four endmembers using the least squares method with the following constraint:

(5) $$\overset{n}{\underset{k=1}{\sum }}f_{k}=1,f_{k}\geq 0$$

The impervious surface fraction was estimated by using the low- and high-albedo fractions and the built-up image extracted by Otsu’s. In the built-up image, pixels with a value of 1 were classified as built-up pixels; on the contrary, pixels with a value of 0 were classified as non-built-up pixels. In the built-up pixels, the impervious surface fraction was equal to the sum of low- and high-albedo fractions; in the non-built-up pixels, the impervious surface fraction was equal to the high-albedo fractions and the low-albedo fraction was classified low-albedo pervious surface fraction. For the low-albedo pervious surface fraction, if pixels had an NDVI value less than 0.2, the soil fraction was estimated by the addition of original soil and low-albedo pervious surface fractions. Otherwise, the vegetation fraction was calculated by the summation of original vegetation and low-albedo pervious surface fractions. A detailed explanation of this process can be found in the work of Xu et al. [1].

### 3.3. Accuracy Assessment

**Figure 3:**
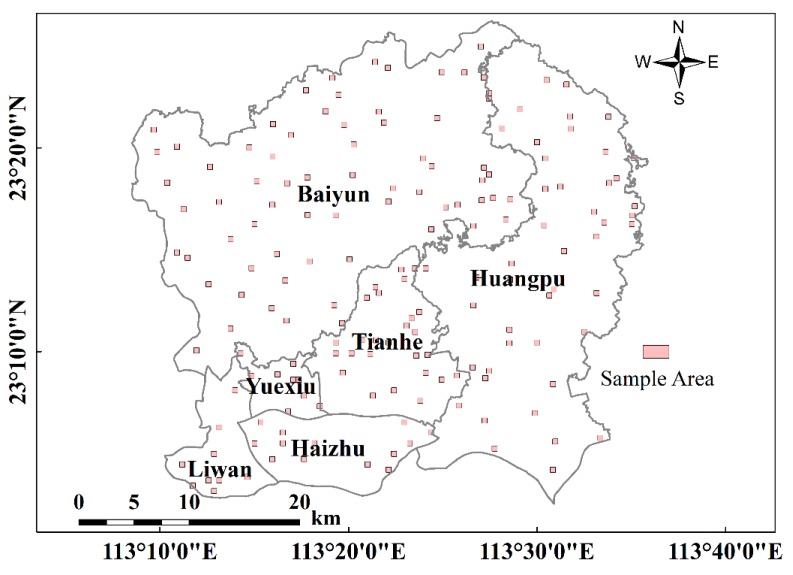
Spatial distribution of the sample areas (shown in pink).

The accuracy of impervious surface extraction was verified with the digitized impervious surface proportion. First, 170 sample areas sized 480 m × 480 m were selected and randomly distributed throughout the study area (Figure 3). Then, the impervious surface in each sample area was digitized on a geometrically-corrected high-resolution image from Google Earth using ArcGIS. The digitized impervious surface proportion was estimated by dividing the digitized areas of impervious surfaces by the sample area, which was regarded as “ground” reference for validating the accuracy of impervious surface extraction. In this study, the root mean square error (RMSE) and bias error (Bias) were used for assessing accuracy.

(6) $$\mathrm{RMSE}=\sqrt{\frac{\sum_{i=1}^{N}{\left({\hat{x}}_{i}-x_{i}\right)}^{2}}{N}}$$

(7) $$\mathrm{Bias}=\frac{\sum_{i=1,}^{N}\left({\hat{x}}_{i}-x_{i}\right)}{N}$$

where ${\hat{x}}_{i}$ is the estimated impervious surface fraction of sample *i* from Sentinel-2A image, $x_{i}$ is the digitized impervious surface proportion from the high-resolution image, and *N* is the number of samples.

## 4. Results

**Figure 4:**
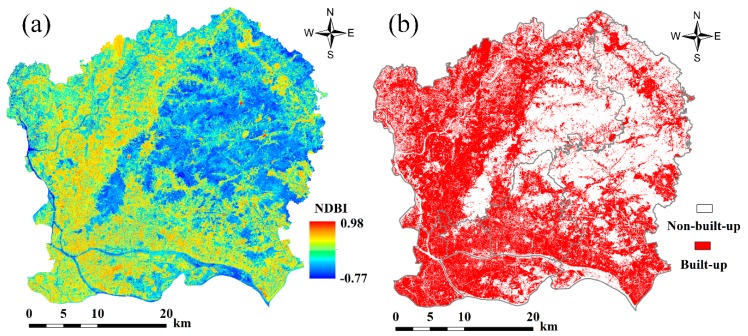
(**a**) Normalized difference built-up index (NDBI; warmer colors indicate a higher index); and (**b**) non-built-up (white) and built-up (red) areas extracted using Otsu’s method.

The NDBI map shown in Figure 4a reveals that the highest NDBI value was 0.98, and the lowest NDBI value was −0.77. High NDBI values were largely found in Liwan, Yuexiu, Haizhu, south Tianhe, southeast Baiyu, and south Huangpu, while low NDBI values were found in west Baiyu and north Huangpu. This pattern likely emerged because forest clusters are mainly located in east Baiyu and north Huangpu. Based on the NDBI image, Otsu’s method was applied to extract urban built-up. The built-up and non-built-up maps can be found in Figure 4b. Figure 4b shows that most built-up areas are gathered around Liwan, Yuexiu, Haizhu, south Tianhe, southeast Baiyu, and south Huangpu, and built-up pixels have high NDBI values. However, the non-built-up areas with low NDBI values are mainly distributed in the west Baiyu and central Huangpu because these areas are mainly covered by forest.

**Figure 5:**
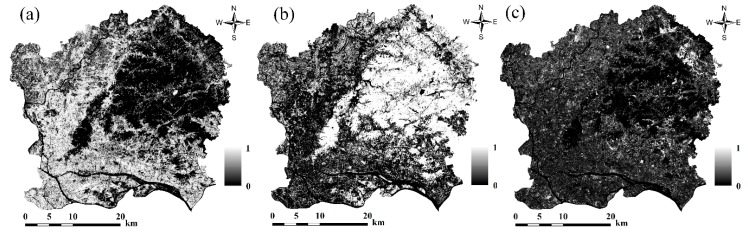
Fraction maps (lighter shades represent increasing representation on a 0–1 scale) extracted from Sentinel-2A imagery via modified LSMA: (**a**) impervious surfaces, (**b**) vegetation, and (**c**) soils.

Based on the built-up image extracted using Otsu’s method, MLSMA was applied to the Sentinel-2A image from 1 November 2017 to extract the impervious surface, vegetation, and soil fractions. The impervious surface, vegetation, and soil maps generated by MLSMA are shown in Figure 5. MLSMA performs well in extracting impervious surface fractions. Most pixels in the forested regions have a vegetation fraction of 100%, an impervious surface fraction of 0%, and a soil fraction of 0%. This is reasonable given that pixels in the forest region have a high vegetation fraction, along with a low impervious surface fraction or an impervious surface fraction of 0%. Figure 5 shows that impervious surface regions are largely found in Liwan, Yuexiu, Haizhu, Tianhe, southwest Baiyu, and south Huangpu while soil can be mainly found in the northeast part of the study area. However, in the built-up areas (Figure 4b), there is still soil fraction of greater than 0% (Figure 4c), which may be unreasonable. In the central urban areas, the land cover types, excluding water bodies, are mainly buildings, roads, and urban squares. Vegetation and bare soil can hardly be found in central urban areas. In general, the built-up pixels of central urban areas are dominantly composed of impervious surfaces and vegetation, and are dominated by impervious surfaces. The apparent errors in the soil fraction may have been introduced by endmember selection. High-albedo and soil objects exhibit similar spectral curves. This may result in high-albedo impervious surfaces mistakenly being treated as soils in LSMA.

**Figure 6:**
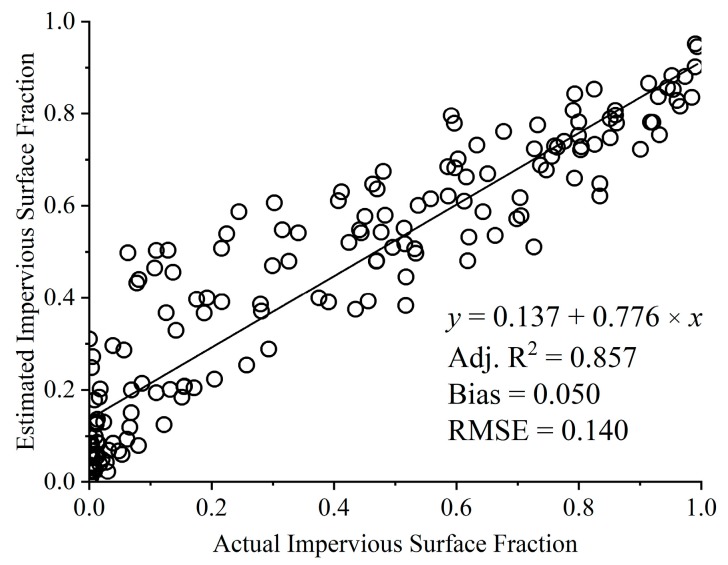
Scatter plot of accuracy assessment results for extracting impervious surfaces from 170 sample areas.

The results of accuracy assessment extracting impervious surfaces from the Sentinel-2A image are shown in Figure 6. The estimated impervious surface fraction of MLSMA is consistent with the actual impervious surface fraction (Figure 6). The RMSE and bias of the impervious surface fraction were 0.140 and 0.050, respectively. There was a large adjusted determination coefficient (Adj. *R*^2^ = 0.857) in the MLSMA. This indicates that MLSMA can be applied to the Sentinel-2A image to extract the impervious surface fraction, and that it does significantly contribute to extract high-precision impervious surface fractions.

**Figure 7:**
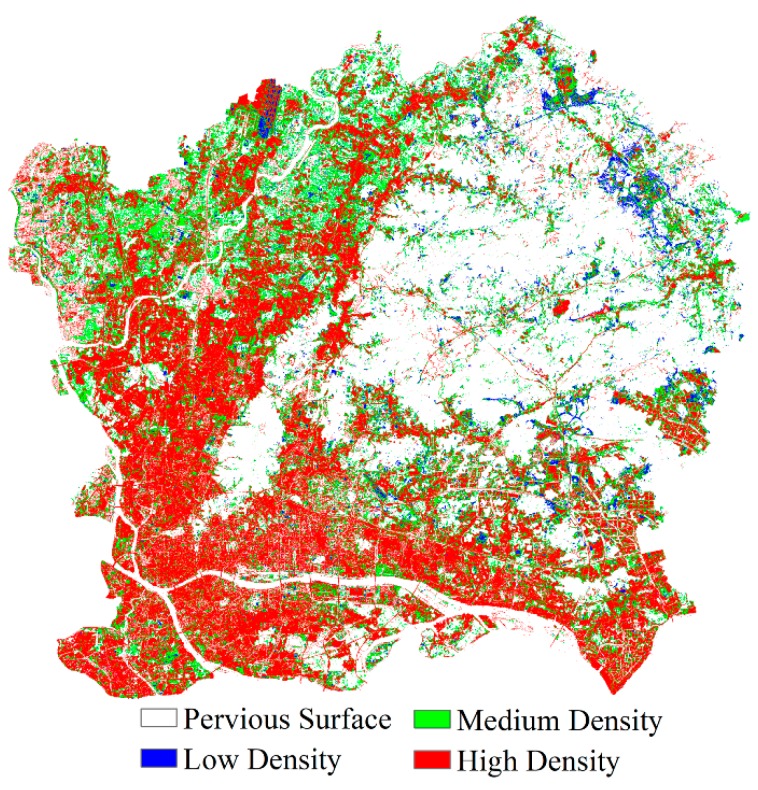
Surface classification map of pervious (white) and impervious surfaces: low density (blue); medium density (green); high density (red).

For the spatial exploration of urban land use, impervious surface fractions were classified into four categories: less than 20% for pervious surfaces, 20–49% for low-density urban lands, 50–79% for medium-density urbans, and 80–100% for high-density urban lands. The classification map of impervious surface fractions is shown in Figure 7. It can be seen from Figure 7 that only a small proportion of low-density pixels were found in the study area, and these were mainly distributed in northern Huangpu. Medium-density pixels were mainly distributed in northwestern Baiyun. High-density pixels were noticeably distributed in Liwan, Yuexiu, Haizhu, southern Baiyun, and southern Tianhe. On the border of Liwan, Yuexiu, and Haizhu, the impervious surface fractions of almost all pixels were greater than 80%. This is reasonable given that Liwan, Yuexiu, and Haizhu comprise the old center of Guangzhou, and contain many buildings, roads and squares. Vegetation was mainly found along roads, and only covered a small area. The soil fraction was less than the vegetation fraction in the central area of Guangzhou. 

## 5. Performance Assessment

**Figure 8:**
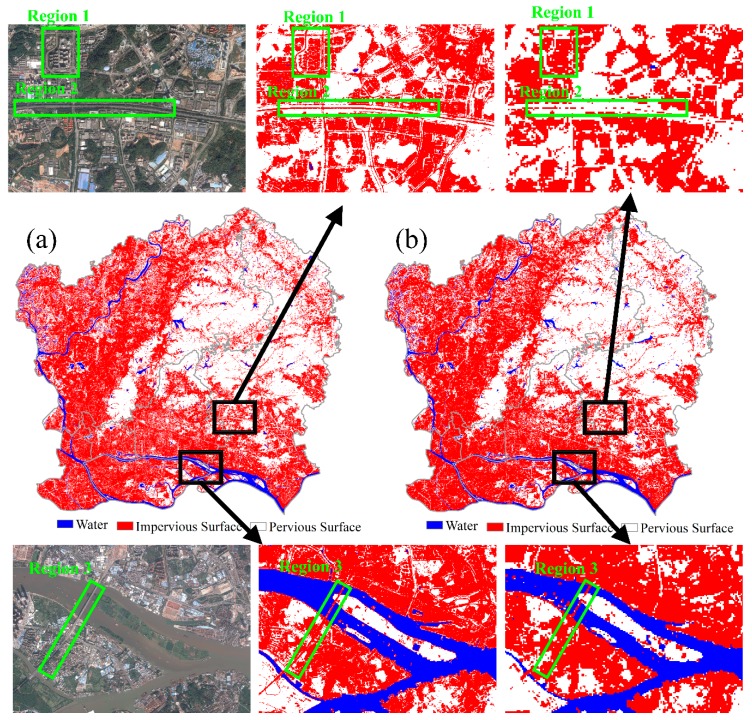
Comparison of impervious surface maps: (**a**) Sentinel-2A, (**b**) Landsat 8.

In this study, an impervious surface fraction map with a spatial resolution of 10 m was extracted from Sentinel-2A image data. A MLSMA method proposed by Xu et al. [1] has been implemented to extract 30 m urban impervious surfaces with a Landsat 8 OLI image acquired on 18 October 2015. The MLSMA was also applied to Sentinel-2A image data to extract 10 m impervious surfaces by integrating Otsu’s method. The extraction performance of impervious surfaces from different remote sensing images with different spatial resolutions was compared with 10 m and 30 m impervious surfaces. The urban impervious surface was mapped with the impervious surface, vegetation, and soil fractions by following methods presented by Jia et al. [63]. The urban impervious surface maps from the Sentinel-2A and Landsat 8 images are shown in Figure 8.

In Figure 8, impervious surfaces are shown in red and pervious surfaces are shown in white; water bodies are shown in blue. It can be seen from Figure 8 that the spatial pattern of the Sentinel-2A impervious surface map is similar to that of the Landsat 8 impervious surface map. Except for forested areas, impervious surfaces have almost covered the entire study area. However, the Sentinel-2A impervious surface map, with its higher spatial resolution, can show more detail than the Landsat 8 impervious surface map (Figure 8).

As shown in regions 1, 2, and 3 of Figure 8, the continuous road segments can be extracted, and the boundaries of buildings can also be clearly identified in the Sentinel-2A impervious surface map. However, it is difficult to extract the continuous road and the boundaries of buildings in the Landsat 8 impervious surface map. This is likely because a pixel in the Landsat 8 image, which has a relatively low spatial resolution of 30 m, may consist of both roads and vegetation, which covers 9 pixels in the Sentinel-2A image due to the latter’s higher spatial resolution. In urban areas, Landsat 8 road pixels were mainly composed of road and vegetation. It is difficult to distinguish pure road pixels from vegetation in this image because the urban road width may be less than 30 m (i.e., the Landsat 8 spatial resolution).

Furthermore, the flourishing plants may cover the road when observed from high-altitude, which may change the reflectance of pure impervious surfaces. In that case, these mixed road pixels may be mistaken as mixed vegetation pixels. This may increase the vegetation fraction and decrease the impervious surface fraction, and introduce errors in mapping impervious surfaces as the impervious surface pixels may be changed to pervious pixels. In contrast, for urban areas, the spatial pattern of roads and buildings with width greater than 10 m can be well recognized by using the Sentinel-2A image.

**Figure 9:**
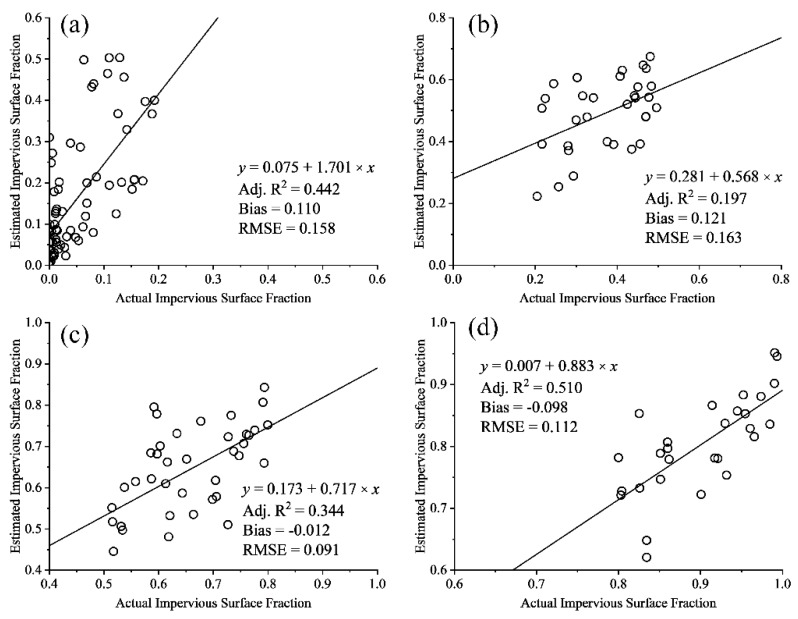
Scatter plot of accuracy assessment results for different impervious surface fractions from 170 sample areas: (**a**) 0–20%, (**b**) 20–49%, (**c**) 50–79%, (**d**) 80–100%.

The high correlation coefficient (Adj. *R*^2^ = 0.857, shown in Figure 6) between the real and estimated fraction of impervious surfaces and low bias (0.05) indicate that MLSMA can be used to extract high-precision impervious surfaces at a 10 m spatial resolution from Sentinel-2A images. However, large errors of magnitude greater than 14% can still be identified in the impervious surface fractions of MLSMA (Figure 6). To further investigate and analyze the errors in impervious surface fractions by urban land use, an accuracy assessment for different impervious surfaces were conducted (Figure 9).

In Figure 9, the impervious surface fraction of MLSMA has large errors in the categories of pervious surfaces and low-density urban lands, whose RMSEs are greater than 0.158. The worst result with the largest bias, RMSE and the minimum Adj. *R*^2^, occurs for low-density urban lands. If the impervious surface fraction is greater than 0.5, MLSMA can be used to extract the high-precision impervious surface fraction. The best result occurs for high-density urban lands, where the Adj. *R*^2^ = 0.510. 

Large errors still exist in the MLSMA results, partly because of the numerical problems of LSMA and the imprecise selection of pure endmembers. For instance, some high-albedo endmember fractions in the pure impervious surface pixels may be misclassified as soil endmembers. During the experiments, we found that the reflectance of the soil endmember was similar to that of the high-albedo endmember over six spectral bands. The spectral curves of endmembers from the Sentinel-2A image are not shown in this study, but are similar to those from the Landsat 8 image (shown in Figure 7 of Xu et al. [1]). This may mistakenly treat the high-albedo fraction as the soil fraction in MLSMA. It also can be seen from Figure 4b that a soil fraction of greater than 0% can still be found in the built-up areas. In practice, the land surface of the central urban areas is mainly covered by buildings, roads, urban square, vegetation, and water bodies. It is unlikely that large areas of bare soil are found in central urban areas. Therefore, to enhance the accuracy of the MLSMA results, a more reasonable MLSMA method could be exploited for extracting the impervious surface fraction with high spatial resolution by combining improved endmember selection and the bare soil index in future.

The soil index was introduced to separate bare soils from impervious surfaces. Based on the bare soil image, the soil fraction was post-processed as the pure soil fraction, and the other soil fraction was treated as the impervious surface fraction. Furthermore, a class-based multiple endmember spectral mixture analysis method proposed by Deng et al. [64] may be useful for extracting high-precision impervious surfaces with Sentinel-2A images. In future research of extracting impervious surface, the Sentinel-2A image may be firstly classified into three land cover classes in the central urban areas: pure impervious surface, pure vegetation, and hybrid impervious surface vegetation; with the information of land cover classes, MLSMA may be then applied to extract the impervious surface fraction.

## 6. Conclusions

The newly available Sentine-2A and -2B data can provide fine spatial resolution multispectral imagery at a 5-day temporal resolution, making it an important dataset for urban expansion monitoring at the regional scale. Our main objective was to explore the extraction performance of high spatial resolution urban impervious surfaces with a Sentinel-2A image. In this study, modified linear spectral mixture analysis (MLSMA) was applied for extracting 10 m impervious surface fractions from the Sentinel-2A image for the main urban area of Guangzhou. The built-up image was first extracted with NDBI using Otsu’s method; the fractions of high-albedo, low-albedo, vegetation and soil were then calculated using conventional LSMA. The results of LSMA were post-processed to estimate the high-precision impervious surface, vegetation, and soil fractions by integrating the built-up image and NDVI. To compare the extraction performance in different spatial resolution images, MLSMA was also applied to a 30 m spatial resolution Landsat 8 OLI image. The results showed that MLSMA could be successfully applied to the 10 m Sentinel-2A image to extract the high-precision impervious surface fraction. The 10 m impervious surface map of Sentinel-2A revealed more detail than the 30 m impervious surface map of Landsat 8 OLI. In the Sentinel-2A impervious surface map, the continuous road and the boundaries of buildings could be clearly identified. However, it was almost impossible for the Landsat 8 OLI image to distinguish these boundaries. 

The bias and correlation coefficient of the 10 m impervious surface fraction were 0.05 and 0.926, respectively. However, RMSEs greater than 0.14 were still found in the MLSMA results. In this study, accuracy assessment was also implemented to analyze the error characteristics of impervious surfaces by different urban land uses. The results showed that MLSMA generally performed well in extracting the high-precision impervious surface fraction if the impervious surface fraction was larger than 0.5. When the impervious surface fraction was less than 0.5, large RMSEs greater than 0.158 were also found in MLSMA results. This may be partly attributed to the least squares approximate solution problems of LSMA and the imprecise selection of pure endmembers. In conclusion, MLSMA can extract the accurate impervious surface fraction for Sentinel-2A MSI imagery. In future research, the improved endmember selection and the bare soil index can be used to improve MLSMA results. Moreover, machine learning classification can be integrated into MLSMA to improve the results obtained in this study.

## Figures and Tables

**Table 1:** Central wavelengths and spatial resolutions of all 13 Sentinel-2A and -2B bands.

Bands	Central Wavelength (mm)	Spatial Resolution (m)
Band 1—Coastal aerosol	0.443	60
Band 2—Blue	0.490	10
Band 3—Green	0.560	10
Band 4—Red	0.665	10
Band 5—Vegetation Red Edge	0.705	20
Band 6—Vegetation Red Edge	0.740	20
Band 7—Vegetation Red Edge	0.783	20
Band 8a—Vegetation Red Edge	0.865	20
Band 8b—NIR	0.842	10
Band 9—Water vapor	0.945	60
Band 10—SWIR/Cirrus	1.375	60
Band 11—SWIR	1.610	20
Band 12—SWIR	2.190	20

## References

[1] Xu J., Zhao Y., Zhong K., Ruan H., Liu X. (2016). Coupling modified linear spectral mixture analysis and soil conservation service curve number (SCS-CN) models to simulate surface runoff: Application to the main urban area of Guangzhou, China. Water.

[2] Yuan F., Bauer M.E. (2007). Comparison of impervious surface area and normalized difference vegetation index as indicators of surface urban heat island effects in landsat imagery. Remote Sens. Environ..

[3] Brabec E., Schulte S., Richards P.L. (2002). Impervious surfaces and water quality: A review of current literature and its implications for watershed planning. J. Plan. Lit..

[4] Weng Q., Lu D., Liang B. (2006). Urban surface biophysical descriptors and land surface temperature variations. Photogramm. Eng. Remote Sens..

[5] Dougherty M., Dymond R.L., Goetz S.J., Jantz C., Goulet N. (2004). Evaluation of impervious surface estimates in a rapidly urbanizing watershed. Photogramm. Eng. Remote Sens..

[6] Xu J., Zhao Y., Zhong K., Zhang F., Liu X., Sun C. (2018). Measuring spatio-temporal dynamics of impervious surface in Guangzhou, China, from 1988 to 2015, using time-series landsat imagery. Sci. Total Environ..

[7] Yu D., Wu C. (2004). Understanding population segregation from landsat ETM+ imagery: A geographically weighted regression approach. Gisci. Remote Sens..

[8] Phinn S.R., Stanford M., Scarth P., Murray A.T., Shyy P.T. (2002). Monitoring the composition of urban environments based on the vegetation-impervious surface-soil (VIS) model by subpixel analysis techniques. Int. J. Remote Sens..

[9] Henits L., Mucsi L., Liska C.M. (2017). Monitoring the changes in impervious surface ratio and urban heat island intensity between 1987 and 2011 in Szeged, Hungary. Environ. Monit. Assess..

[10] Li L., Lu D., Kuang W. (2016). Examining urban impervious surface distribution and its dynamic change in Hangzhou metropolis. Remote Sens..

[11] Yang L., Huang C., Homer C.G., Wylie B.K., Coan M. (2003). An approach for mapping large-area impervious surfaces: Synergistic use of landsat-7 ETM+ and high spatial resolution imagery. Can. J. Remote Sens..

[12] Piyoosh A.K., Ghosh S.K. (2017). Semi-automatic mapping of anthropogenic impervious surfaces in an urban/suburban area using Landsat 8 satellite data. Gisci. Remote Sens..

[13] Deng C., Li C., Zhu Z., Lin W., Xi L. (2017). Subpixel urban impervious surface mapping: The impact of input landsat images. ISPRS J. Photogramm. Remote Sens..

[14] Zhang L., Weng Q., Shao Z. (2017). An evaluation of monthly impervious surface dynamics by fusing Landsat and MODIS time series in the Pearl River Delta, China, from 2000 to 2015. Remote Sens. Environ..

[15] Cablk M.E., Minor T.B. (2003). Detecting and discriminating impervious cover with high-resolution IKONOS data using principal component analysis and morphological operators. Int. J. Remote Sens..

[16] Lu D., Weng Q. (2009). Extraction of urban impervious surfaces from an IKONOS image. J. Remote Sens..

[17] Yang J., Li P. (2015). Impervious surface extraction in urban areas from high spatial resolution imagery using linear spectral unmixing. Remote Sens. Appl. Soc. Environ..

[18] Weng Q., Hu X., Liu H. (2009). Estimating impervious surfaces using linear spectral mixture analysis with multitemporal aster images. Int. J. Remote Sens..

[19] Mallick J., Rahman A., Singh C.K. (2013). Modeling urban heat islands in heterogeneous land surface and its correlation with impervious surface area by using night-time ASTER satellite data in highly urbanizing city, Delhi-India. Adv. Space Res..

[20] Yao Y., He J., Zhang J., Zhang Y. (2017). Extracting urban impervious surface from GF-1 imagery using one-class classifiers. arXiv.

[21] Zhang H., Lin H., Li Y., Zhang Y., Fang C. (2016). Mapping urban impervious surface with dual-polarimetric SAR data: An improved method. Lands. Urban Plan..

[22] Zhang H., Lin H., Wang Y. (2018). A new scheme for urban impervious surface classification from SAR images. ISPRS J. Photogramm. Remote Sens..

[23] Tison C., Nicolas J., Tupin F., Maitre H. (2004). A new statistical model for Markovian classification of urban areas in high-resolution SAR images. Int. Geosci. Remote Sens. Symp..

[24] Shao Z., Liu C. (2014). The integrated use of DMSP-OLS nighttime light and MODIS data for monitoring large-scale impervious surface dynamics: A case study in the yangtze river delta. Remote Sens..

[25] Guo W., Lu D., Kuang W. (2017). Improving fractional impervious surface mapping performance through combination of DMSP-OLS and MODIS NDVI data. Remote Sens..

[26] Fu H., Shao Z., Tu C., Zhang Q. Impacts of Feature Selection for urban Impervious Surface Extraction Using Optical Image and SAR Data. Proceedings of the International Workshop on Earth Observation and remote Sensing Applications.

[27] Yang L., Jiang L., Lin H., Liao M. (2009). Quantifying sub-pixel urban impervious surface through fusion of optical and inSAR imagery. Mapp. Sci. Remote Sens..

[28] Zhang Y., Zhang H., Lin H. (2014). Improving the impervious surface estimation with combined use of optical and SAR remote sensing images. Remote Sens. Environ..

[29] Zhang H., Xu R. (2018). Exploring the optimal integration levels between SAR and optical data for better urban land cover mapping in the Pearl River Delta. Int. J. Appl. Earth Obs. Geoinf..

[30] Deyong H.U., Chen S., Qiao K., Cao S. (2017). Integrating CART algorithm and multi-source remote sensing data to estimate sub-pixel impervious surface coverage: A case study from Beijing municipality, China. Chin. Geogr. Sci..

[31] Weng Q., Hu X. (2008). Medium spatial resolution satellite imagery for estimating and mapping urban impervious surfaces using LSMA and ANN. IEEE Trans. Geosci. Remote Sens..

[32] Sun G., Chen X., Ren J., Zhang A., Jia X. (2017). Stratified spectral mixture analysis of medium resolution imagery for impervious surface mapping. Int. J. Appl. Earth Obs. Geoinf..

[33] Demarchi L., Canters F., Chan J.C., De Voorde T.V. (2012). Multiple endmember unmixing of CHRIS/Proba imagery for mapping impervious surfaces in urban and suburban environments. IEEE Trans. Geosci. Remote Sens..

[34] Li W., Wu C. (2016). A geostatistical temporal mixture analysis approach to address endmember variability for estimating regional impervious surface distributions. Gisci. Remote Sens..

[35] Yu X., Shen Z., Cheng X., Xia L., Luo J. (2016). Impervious surface extraction using coupled spectral–spatial features. J. Appl. Remote Sens..

[36] Wu C., Murray A.T. (2003). Estimating impervious surface distribution by spectral mixture analysis. Remote Sens. Environ..

[37] Weng Q., Hu X., Lu D. (2008). Extracting impervious surfaces from medium spatial resolution multispectral and hyperspectral imagery: A comparison. J. Remote Sens..

[38] Yang J., He Y. (2017). Automated mapping of impervious surfaces in urban and suburban areas: Linear spectral unmixing of high spatial resolution imagery. Int. J. App. Earth Obs. Geoinf..

[39] Goetz S.J., Wright R., Smith A., Zinecker E., Schaub E. (2003). IKONOS imagery for resource management: Tree cover, impervious surfaces, and riparian buffer analyses in the mid-atlantic region. Remote Sens. Environ..

[40] Der Linden S.V., Hostert P. (2009). The influence of urban structures on impervious surface maps from airborne hyperspectral data. Remote Sens. Environ..

[41] Mayes M., Mustard J.F., Melillo J.M. (2015). Forest cover change in miombo woodlands: Modeling land cover of African dry tropical forests with linear spectral mixture analysis. Remote Sens. Environ..

[42] Chen Y., Yu S. (2016). Assessment of urban growth in Guangzhou using multi-temporal, multi-sensor landsat data to quantify and map impervious surfaces. J. Remote Sens..

[43] Thouvenin P., Dobigeon N., Tourneret J. (2016). Hyperspectral unmixing with spectral variability using a perturbed linear mixing model. IEEE Trans. Signal Process..

[44] Ma L., Chen J., Zhou Y., Chen X. (2016). Two-step constrained nonlinear spectral mixture analysis method for mitigating the collinearity effect. IEEE Trans. Geosci. Remote Sens..

[45] Li W., Wu C. (2015). Incorporating land use land cover probability information into endmember class selections for temporal mixture analysis. ISPRS J. Photogramm. Remote Sens..

[46] Fan F., Fan W., Weng Q. (2015). Improving urban impervious surface mapping by linear spectral mixture analysis and using spectral indices. Can. J. Remote Sens..

[47] Drusch M., Bello U.D., Carlier S., Colin O., Fernandez V., Gascon F., Hoersch B., Isola C., Laberinti P., Martimort P. (2012). Sentinel-2: ESA’s optical high-resolution mission for GMES operational services. Remote Sens. Environ..

[48] Hagolle O., Sylvander S., Huc M., Claverie M., Clesse D., Dechoz C., Lonjou V., Poulain V. (2015). SPOT-4 (Take 5): Simulation of Sentinel-2 time series on 45 large sites. Remote Sens..

[49] Segl K., Guanter L., Gascon F., Kuester T., Rogass C., Mielke C. (2015). S2eteS: An end-to-end modeling tool for the simulation of Sentinel-2 image products. IEEE Trans. Geosci. Remote Sens..

[50] Immitzer M., Vuolo F., Atzberger C. (2016). First experience with Sentinel-2 data for crop and tree species classifications in central europe. Remote Sens..

[51] Lefebvre A., Sannier C., Corpetti T. (2016). Monitoring urban areas with Sentinel-2A data: Application to the update of the copernicus high resolution layer imperviousness degree. Remote Sens..

[52] Pesaresi M., Corbane C., Julea A., Florczyk A.J., Syrris V., Soille P. (2016). Assessment of the added-value of Sentinel-2 for detecting built-up areas. Remote Sens..

[53] Yang X., Zhao S., Qin X., Zhao N., Liang L. (2017). Mapping of urban surface water bodies from Sentinel-2 MSI imagery at 10 m resolution via NDWI-based image sharpening. Remote Sens..

[54] Du Y., Zhang Y., Ling F., Wang Q., Li W., Li X. (2016). Water bodies’ mapping from Sentinel-2 imagery with modified normalized difference water index at 10-m spatial resolution produced by sharpening the SWIR band. Remote Sens..

[55] Zha Y., Gao J., Ni S. (2003). Use of normalized difference built-up index in automatically mapping urban areas from tm imagery. Int. J. Remote Sens..

[56] Varshney A., Rajesh E. (2014). A comparative study of built-up index approaches for automated extraction of built-up regions from remote sensing data. J. Indian Soc. Remote Sens..

[57] Otsu N. (1979). A threshold selection method from gray-level histograms. IEEE Trans. Syst. Man Cybern..

[58] Liu X., Deng R., Xu J., Zhang F. (2017). Coupling the modified linear spectral mixture analysis and pixel-swapping methods for improving subpixel water mapping: Application to the Pearl River Delta, China. Water.

[59] Du Z., Li W., Zhou D., Tian L., Ling F., Wang H., Gui Y., Sun B. (2014). Analysis of landsat-8 OLI imagery for land surface water mapping. Remote Sens. Lett..

[60] Xie H., Luo X., Xu X., Pan H., Tong X. (2016). Automated subpixel surface water mapping from heterogeneous urban environments using Landsat 8 OLI imagery. Remote Sens..

[61] Hanqiu X.U. (2005). A study on information extraction of water body with the modified normalized difference water index (MNDWI). J. Remote Sens..

[62] Adams J.B., Sabol D.E., Kapos V., Filho R.A., Roberts D.A., Smith M.O., Gillespie A.R. (1995). Classification of multispectral images based on fractions of endmembers: Application to land-cover change in the Brazilian Amazon. Remote Sens. Environ..

[63] Jia Y., Tang L., Wang L. (2017). Influence of ecological factors on estimation of impervious surface area using Landsat 8 imagery. Remote Sens..

[64] Deng Y., Wu C. (2016). Development of a class-based multiple endmember spectral mixture analysis (C-MESMA) approach for analyzing urban environments. Remote Sens..

